# Photoluminescent carbon dots (PCDs) from sour apple: a biocompatible nanomaterial for preventing UHMWPE wear-particle induced osteolysis via modulating Chemerin/ChemR23 and SIRT1 signaling pathway and its bioimaging application

**DOI:** 10.1186/s12951-022-01498-3

**Published:** 2022-06-27

**Authors:** Xiang Li, Yang Lu, Jiarui Li, Shengji Zhou, Yuxin Wang, Liangping Li, Fengchao Zhao

**Affiliations:** 1 Department of Orthopaedic Surgery, The First Affiliated Hospital, Zhejiang University School of Medicine, No.79 Qingchun Road, Hangzhou, 310003 People’s Republic of China; 2 Department of Surgery, The Second Affiliated Hospital of Zhejiang University School of Medicine, Hangzhou, 310003 People’s Republic of China

**Keywords:** Photoluminescent carbon-dots, Osteolysis, Mouse-calvarial model, Chemerin-ChemR23 signaling, SIRT1 pathway

## Abstract

Photoluminescent nanomaterials have been widely employed in several biological applications both in vitro and in vivo. For the first time, we report a novel application of sour apple-derived photoluminescent carbon dots (PCDs) for reducing ultra-high molecular weight polyethylene (UHMWPE) wear particle-induced osteolysis using mouse calvarial model. Generally, aseptic prosthetic loosening seems to be a significant postoperative problem for artificial joints replacement, which is mainly contributed by UHMWPE-induced osteolysis. Hence, inhibiting osteoclastic bone-resorption could minimize UHMWPE-induced osteolysis for implant loosening. Prior to osteolysis studies, the prepared sour apple-derived PCDs were employed for bioimaging application. As expected, the prepared PCDs effectively inhibited the UHMWPE particle-induced osteoclastogenesis in vitro. The PCDs treatment effectively inhibited the UHMWPE-induced osteoclast differentiation, F-actin ring pattern, and bone resorption in vitro. Also, the PCDs reduced the UHMWPE-induced ROS stress as well as the expression level of pro-inflammatory cytokines, including TNF-α, IL-1, IL-6, and IL-8. Further, the qPCR and western blot results hypothesized that PCDs inhibited the UHMWPE wear particle-induced osteolysis through suppressing chemerin/ChemR23 signaling and NFATc1 pathway, along with upregulation of SIRT1 expression. Overall, these findings suggest that the synthesized PCDs could be a potential therapeutic material for minimizing UHMWPE particle-induced periprosthetic osteolysis to avoid postoperative complications.

**Supplementary Information:**

The online version contains supplementary material available at 10.1186/s12951-022-01498-3.

## Introduction

In a scenario wherein arthroplasties are progressively having operated on transplant patients, osteolysis is the major cause of errors during joint replacement prosthesis has received much interest [1]. Wear particle-induced osteolysis caused by ultra-high molecular weight polyethylene has been the practice through which reconstructive wreckage statically released from the substratum of prosthetic joint surfaces provokes an inflammatory system that favors bone catabolism, culminating in weakening of prosthetics and imminent downfall or cracking [2, 3]. Wear particles made of ultra-high molecular weight polyethylene (UHMWPE) have now been identified as among the most common causes of aseptic loosening in total orthopedic implants [4]. However, UHMWPE has been widely employed as a prosthetic material for complete orthopedic implants because of its superior biocompatibility, low contact-pressure, and anti-corrosion properties [5]. The artificial wear particles in the interstitial bone/prosthesis membrane trigger macrophages and fibroblasts in the implants region, resulting in osteolysis [6].

In the fundamentals of prosthesis loosening, the stimulation of macrophages, as well as foreign-body giant cells and the phagocytosis of UHMWPE-particle wear debris, initiates osteolysis [7]. Recent studies have proved that macrophages mainly contribute to symptomatic osteolysis by vigorously phagocytozing wear particles and producing inflammatory responses, resulting in an unbalanced osteoclastogenesis and process of bone growth [4]. Wear particles stimulate the secretion of pro-inflammatory cytokines and growth factors in osteoporotic tissues that acquire and polarize macrophages and osteoclast progenitor cells [8]. Afterward, the stimulated macrophages enhance osteoclast formation, and restrict osteoblast differentiation and function, resulting in prosthesis loosening [9].

The processes of UHMWPE particle-induced osteolysis are complicated and include a number of mechanisms [10, 11]. In brief, the hematopoietic bloodline cells produce multinucleated large bone-destructing cells called osteoclasts [12]. It is well-known that osteoclasts cause osteolysis, which leads to aseptic loosening [13]. The osteoclast-stimulated cytokines such as macrophage colony-stimulating factor (M-CSF) and receptor activator of nuclear factor kappa B (NF-κB) ligand are involved in osteoclastogenesis by the stimulation of osteoclastic signaling pathways including MAPK, NF-κB, and NFATc1 signaling pathways, leads to the increase of c-fos and NFATc1 production, resulting in osteoclastogenesis [14–16]. Furthermore, the Chemerin/ChemR23 signaling pathway has important roles in modulating the actions of UHMWPE-particles on osteoblast and osteoclast development during osteolysis [17]. On other hand, suppressing the SIRT1 (Sirtuin 1, a nicotinamide adenine dinucleotide (NAD)-dependent deacetylase) expression also enhance the osteoclastogenesis [18]. As a result, inhibiting these osteoclast-associated signaling pathways might be an alternate treatment for UHMWPE particle-stimulated aseptic prosthetic loosening [12].

Osteolysis has long been considered a "surgical illness". Surgery is required to replacing the failing articulation and to repair any earlier or current bone loss [19]. In non-operative treatment, promoting osteoclast apoptosis, suppressing osteoclastogenesis, and hindering bone resorption are the three techniques available for fighting osteoclast-related problems [20]. Bisphosphonates are generally recommended for the treatment of osteolysis related bone diseases, which are targeting osteoclast by promoting osteoclast apoptosis and hindering bone resorption. Furthermore, numerous compounds such as amentoflavone [21], anthocyanin [22], enalapril [23], geraniin [24], resveratrol [25], hydrogen sulfide [26], melatonin [27], notoginsenoside R1 [28], and others have been showed their potential in mouse calvarial model. In recent, [29] reported that chitosan-derived carbon dots suppress osteolysis and lipopolysaccharide-induced calvarial bone destruction in mice. In the present study, we report a novel sour apple-derived photoluminescent carbon dots (PCDs) for reducing UHMWPE particle-induced osteolysis in mouse calvarial model.

PCDs are carbonic nanomaterials with a quasi-spherical configuration (usually below 10 nm in size) with a nanocrystalline structure of graphitic sp2 carbon atoms and sp3 carbon defects [30]. PCDs have exceptional physicochemical and biological features, making them ideal for use in biomedical science [31–33]. PCDs have gained popularity in nanobiotechnology because of their exceptional photostability, rising water solubility, simplicity of synthesis and surface fabrication, inertness, non-toxicity, and outstanding biocompatibility [34]. PCDs also have strong two-photon absorption and unique tunable optical characteristics more than a broad range of frequencies, from ultraviolet to near infrared, making it suitable for a wide range of biomedical applications [35]. Furthermore, CQDs are well-known fluorescent materials that can be used in a variety of applications, including bioimaging, metal sensing, photocatalytic reactions, opto-electronic usages, and photopolymerization application [36–38]. Notably, the excellent biocompatibility, effortless clearance from the body, inexpensive, and immune system obfuscation are all characteristics that make carbon dot an excellent candidate for bio-labelling and bioimaging applications [39–41]. Several studies have been reported the multifunctional applications of CQDs including antimicrobial [42], antibiofilm [43], antifogging [44], antioxidant activity [45], and other biomedical applications [32] [46, 47]. The surface functionalized PCDs with anticancer drugs have great interest due to the delivery of nanomedicine at target site in the field of chemotherapy [48, 49].

In the present study, we reported the inhibitory potential of sour apple peel-extracted PCDs against UHMWPE particle-induced osteolysis in mouse calvarial model. This will be the first study to demonstrate the rescue action of the biosynthesized PCDs on UHMWPE particle-induced osteolysis for treating postoperative complications. The protective effect of PCDs on UHMWPE-induced osteoclast differentiation, F-actin ring pattern, and bone resorption was examined. Furthermore, the effects on UHMWPE-induced ROS stress and the expression level of pro-inflammatory cytokines (TNF-α, IL-1, IL-6, and IL-8) were studied. On the other hand, the in vivo fluorescence of PCDs was assessed via zebrafish model for bioimaging application.

## Materials and methods

### Preparation and characterization of photoluminescence carbon dots (PCDs)

In the present study, photoluminescence carbon dots (PCDs) were prepared using peal extract of sour apple as carbon source through hydrothermal method. For the experiment, fresh sour apples were purchased from a local market at Hangzhou, China. The apple peels were dried in dark condition for 4 days and the dried peels were ground to a fine powder. Then, the peel powder was dispersed into 100 mL of distilled water and heated at 60 ºC for 3 h. Subsequently, the collected aqueous extract was transferred to a 200 mL Teflon-lined autoclave and heated at 180 ºC for 4 h under hydrothermal condition for the carbonization process. After, the fluorescent carbon dots in supernatant were separated via centrifugation at 10,000 rpm for 15 min. Finally, the supernatant was filtered using 0.2 µM filter membrane. Then, the small molecules were removed through dialysis using a dialysis membrane of 10 k molecular weight cut-off. Finally, the filtered-solution of PCDs was lyophilized for subsequent studies. For compositional characterization, the prepared PCDs were allowed to HR-TEM with SEAD, XRD, FT-IR, and XPS analysis. In order to assess the optical properties of PCDs, UV-Vis spectroscopic and photoluminescence spectroscopic analysis were performed. Quantum yield of the sour apple-derived PCDs were measured through comparative method using quinine sulfate as a standard [50].

### Assessing toxicity level of PCD using zebrafish model

Adult zebrafish (*Danio rerio*) (> 8 month old, Wild type) were obtained from an aquaculture farm at Hangzhou, China. The collected zebrafishes were acclimatized at 28 ± 2 °C for seven days in a glass aquarium containing filtered fresh water and fed with commercial food pellets twice a day. All adult fish were maintained with a 12 h photoperiod at 25 ºC under 40 L glass aquaria with aerated recirculating water. The water quality was checked throughout the experiment. During the experiment, the *Artemia franciscana* nauplii were provided as food. Subsequently, the healthy adult female fish were differentiated and separated from the male fish by their larger abdomen, more yellowish color, and the presence of a genital papilla. For spawning, healthy adult zebrafish with high potential to produce fertilized eggs were kept in separate 2 L aquarium tanks with the recommended water quantity of 1 L per fish and kept in 10 h/14 h dark and light periods. The fish were spawned successfully. The viable embryos produced were collected and placed in separate aquaria and held for 4 days before starting the toxicity experiment. Then, the healthy 4 days larvae were collected and used for the larvae-mediated toxicity experiments [51]. For toxicity assay, ten healthy zebrafish-larvae were transferred to wells of a 24-well microtiter plate containing the different concentrations of PCD in water, and the plates were incubated at 25 ºC for 24 h. The mortality was counted every 1 h and the survival percentage was calculated [52].

### Bioimaging application of PCDs

For bioimaging application, zebrafish larvae were incubated in water containing PCDs for 1 h. After incubation, the stained animals were washed three times with PBS. Finally, the in vivo fluorescence of PCD was detected and imaged using fluorescence microscope (BX-53 Olympus™) [53]. For bacterial biofilm imaging, *Staphylococcus aureus* (ATCC 25,923) cells were grown in Tryptic soy broth (TSB) in 24 well microtiter plate for 24 h at 37 ºC. In this assay, the bacterial cells were allowed to form biofilm on glass slides (1 × 1 cm) [54]. After 24 h, the glass slides were washed with distilled water and stained with PCDs solution for 10 min. Finally, the slides were imaged using fluorescence microscope [55].

### Assessing the antioxidant property of PCDs

The antioxidant behavior of prepared PCDs was examined through DPPH scavenging assay as described in [26]. Likewise, the antioxidant potentials of PCDs to scavenge other biological ROS radicals such as H_2_O_2_, •OH, and O_2_• − radicals were determined using H_2_O_2_ scavenging assay [56], hydroxyl radical scavenging assay [57], and singlet oxygen quenching assay [58], respectively.

### Bone marrow-derived macrophages (BMMs) isolation and osteoclasts culture

BMMs were collected from the femoral condyles of two-month-old healthy male C57BL/6 J mice to isolate and cultivate the osteocytes progenitor cells. To cultivate the BMMs, HyClone α-minimum essential medium (α-MEM) with M-CSF (10 ng/mL) was used. Cell suspensions were taken the next day, centrifuged, and reintroduced in α-MEM containing M-CSF (30 ng/mL). After 72 h of culturing, the osteoclastic precursor cells were harvested and stored for subsequent studies.

### Toxicity assessment on BMMs

Toxicity level of PCDs on BMMs was examined by measuring the cell viability using Cell Counting Kit-8 (CCK-8) assay [59]. For this experiment, BMMs cells (2 × 10^4^ cells/well) were incubated with α-MEM containing M-CSF (30 ng/mL) for 24 h. Then, the BMMs were reintroduced in α-MEM containing M-CSF (30 ng/mL) and different concentrations of prepared PCD and incubated for 48 h. After, the BMMs were incubated with 10% CCK-8 containing α-MEM for 3 h and the cell viability was measured at 450 nm using a multilabel-plate reader.

### Effect of PCDs on UHMWPE-induced osteoclast differentiation

For the experiment, UHMWPE wear particles (53–75 μm in diameters) were acquired from Sigma-Aldrich, USA. In the present study, BMMs were utilized to evaluate the capacity of osteoclasts to generate new osteoporosis. The BMMs cells were grown and stimulated in α-MEM medium with M-CSF (30 ng/mL), UHMWPE wear particles (1 mg/mL), and various concentrations of PCDs for 7 days. After incubation, the grown cells were fixed and washed, then allowed to Tartrate-resistant acid phosphatase (TRAP) for examining the osteoclast differentiation from the osteoclastic precursor cells. Then, a light microscope was used to count TRAP-positive cells with more than three nuclei, which were thought to be conventional osteoclasts. The count of osteoclasts was done with ImageJ software.

### F-actin ring staining

To establish the inhibiting impact of PCDs on mature F-actin ring formation in osteoclasts, phalloidin-DAPI staining was used. As-mentioned above, after the osteoclast differentiation with M-CSF (30 ng/mL), UHMWPE wear particles (1 mg/mL), and PCDs (75 µg/mL) for 7 days, cells were fixed with 4% paraformaldehyde and permeabilized with 0.1% Triton-X for 10 min. Then, the cells were stained with phalloidin (diluted in 0.2% (w/v) PBS) for 10 min and the cells were washed, allowed to DAPI staining for 5 min, and visualized under fluorescence microscope (ZEISS Axioscope 5, ZEISS, Germany). Finally, the count of F-actin rings was done with ImageJ software [60].

### Bone resorption pits assay

The possible influence of PCDs on osteoclastic bone resorption pits was investigated using an osteo assay plate (Corning, USA). The cells were transferred to osteo assay plate and grown in α-MEM medium with M-CSF (30 ng/mL), UHMWPE wear particles (1 mg/mL), and PCDs (75 µg/mL) for 7 days. Then, the cells were removed by sonication for visualization with SEM (Hitachi SU9000) and the area of resorption pits were analyzed using Image-J software [27].

### Intracellular ROS measurement

The scavenging effect of PCDs on UHMWPE-induced ROS stress on BMMs was studied using DCFH-DA staining [61]. For ROS measurement, BMMs were grown in α-MEM medium with M-CSF (30 ng/mL), UHMWPE wear particles (1 mg/mL), and PCDs (75 µg/mL) for 3 days. Then, the BMMs were stained with 10 μM of DCFH-DA and visualized under fluorescence microscope. The intracellular ROS level was measured using a ROS assay kit (Beyotime Technology Inc, China).

### qPCR analysis

For qPCR analysis, BMMs were grown in α-MEM medium with M-CSF (30 ng/mL), UHMWPE wear particles (1 mg/mL), and PCDs (75 µg/mL) for 3 days. Then, TRIzol reagent was used to extract total RNA and HiScript-III-RT SuperMix (Vazyme, China) was used to synthesis cDNA. The SYBR Premix Ex Tag kit and the ABI 7500 Sequencing Detection System.

(Applied Biosystems, USA) were utilized to perform the real-time PCR analysis. The housekeeping gene β-actin has been used to normalise all reactions, which were performed in triplicate. Tthe primer sequences were given in Additional file 1: Table S1.

### Western blot analysis

For western blot analysis, BMMs were grown in α-MEM medium with M-CSF (30 ng/mL), UHMWPE wear particles (1 mg/mL), and PCDs (75 µg/mL) for 3 days. Total protein was collected by RIPA lysis buffer (Solarbia, China) containing protease and phosphatase inhibitors. Then, the total protein concentrations were measured using BCA protein assay kit (Thermo Fisher Scientific, USA). After that, the total protein was separated using a 10% SDS gel electrophoresis and then transferred to polyvinylidene fluoride membranes. Membranes were stopped and treated with antibodies at 4 °C overnight, then washed three times and probed with secondary antibodies with 1 h. Next, an expanded chemiluminescence probe (Millipore, USA) was used to generate the protein bands. Finally, the Tanon 5200 system (Bio Tanon, China) was used to monitor antibody interaction, and Image-J software was used to investigate signal strengths. Antibodies of the following were used in this study: Chem (Rarres2), ChemR23, NFATc1, Itgb3, Acp5, CtsK, SIRT1, and β-actin.

### Immunofluorescence staining of SIRT1 expression

In order to identify SIRT1 expression in BMMs, the cells were incubated with anti-SIRT1 antibody at 4 °C overnight. Then, the secondary antibody (goat anti-rabbit IgG) was administered and incubated at 37 °C for 60 min. Finally, the cells were stained with DAPI and observed under a fluorescence microscope [26].

### Effect of PCD in UHMWPE-induced osteolysis in mouse calvarial model

The Animal Experimental Ethical Inspection Committee of the First Affiliated Hospital, College of Medicine, Zhejiang University was approved the animal experiment (Ref. No: 2020-1543). For this study, healthy C57BL/6 J adult mice (7–9 weeks old) were acquired from the Zhejiang Experimental Animal Center. In the experiment, the mice were anaesthetized and 1 cm length incision was made in midline of mouse head and then the periosteum was separated from calvaria. Subsequently, 20 mg UHMWPE-particles were evenly planted over the calvaria. After the wounds subsided, the animals (n = 6) were randomly allocated to one of three groups: sham control, UHMWPE-particles control, and PCDs treated group (the treatment group received UHMWPE-particles and PCDs (7.5 mg/kg) was injected locally). After 14 days, the experimental animals were slaughtered for micro-computed tomography (CT) scanning (Bruker micro-CT, Germany) at a resolution of 9 mm. At last, the bone volume against tissue volume (BV/TV) and total porosity were analyzed using Bruker CT analyzer software (V 1.15.4.0) [3].

### Statistical analysis

All the experiments were done in triplicates. The values were expressed as mean ± SD. The statistical analyses were performed using IBM SPSS Statistics V23.0 (SPSS Ltd, Hong Kong) software package. A Dunnett's-ANOVA test and Student t-Test were used to compare the treated groups with respective controls.

## Results and discussion

### Synthesis and characterization of PCDs

**Scheme 1:**
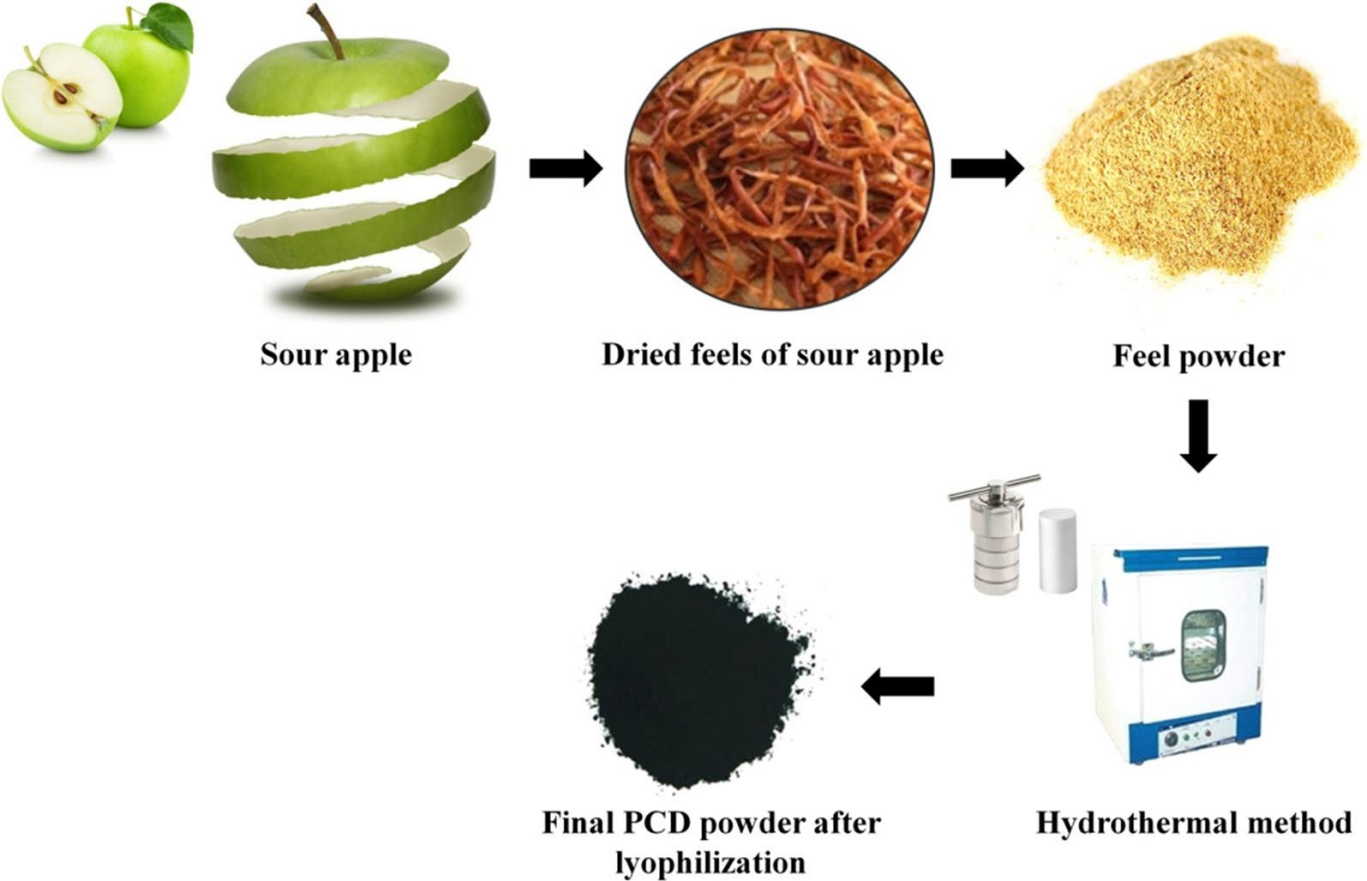
Graphical representation illustrating the preparation of the photoluminescent carbon dots (PCDs) from sour apple feel under hydrothermal method

In the present study, we have prepared photoluminescent carbon dots using sour apple feel extract as carbon and nitrogen source under hydrothermal condition, as mentioned in Scheme 1. Since apple peels are readily available (and usually thrown as waste), the entire methodology of synthesizing PCDs for anti-osteolysis and bioimaging applications is exceedingly cost-effective. The GC-MS-based phytochemical analysis suggests that the major compounds of sour apple peel are malic acid, citric acid, hexadecanoic acid, 9-octadecenoic acid, and propenone,3-(2-benzoxazolylthio)-1-phenyl (Additional file 1: Fig. S1). Hence, it was assume that when peel extracts are destroyed at high temperatures, intermolecular and/or intramolecular decomposition occurs in the compounds of apple peel, resulting in aromatic sp^2^ carbon production through carbonization, and aromatization and polymerization during hydrothermal condition.

**Fig. 1:**
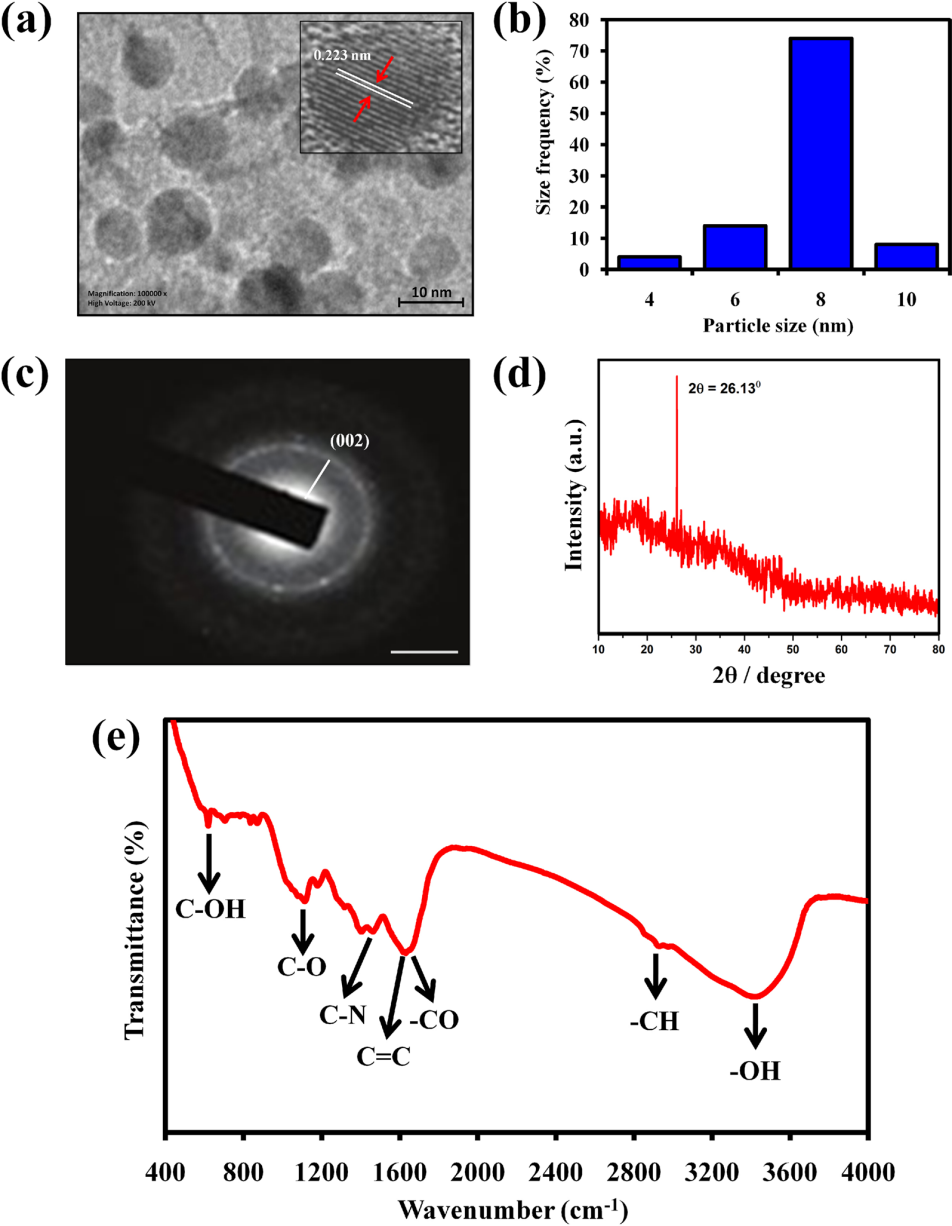
**a** HR-TEM images of the structural characterization of PCDs (Inset represents the lattice d-spacing of 0.347 nm revealing plane of sp.^2^ carbon. **b** Graph representing the size of the synthesized PCDs. **c** SAED pattern of individual material of the PCDs. **d** XRD pattern of the PCDs illustrated a (002) peak authenticating the graphitic nature of the PCDs. **e** FT-IR analysis of the functional groups presented in the surface of PCDs

In Fig. 1a, the HR-TEM picture revealed that the prepared PCDs are spherical crystalline with a lattice spacing of 0.223 nm, confirming the (002) diffraction plane of graphite (sp2) carbon. The synthesized materials are predominantly 8 nm in size, according to DLS analysis (Fig. 1b). The SAED pattern illustrates diffused rings signifying the polycrystalline nature of carbon dots (Fig. 1c). The XRD spectrum of sour apple peel extract showed that the three diffraction peaks at 28.52°, 40.88° and 50.93° are indicating the presence of ascorbic acid (JCPDS 22-1560), citric acid (JCPDS 22-1568), and malic acid (JCPDS 23-1631) in the peel extract (Additional file 1: Fig. S2). This result further corroborates the phytochemical analysis of peel extract identified by GC-MS analysis (Additional file 1: Fig. S1). In Fig. 1d, the XRD pattern of PCDs showed a broad (002) peak at 26.134º 2θ, which symbolizing the graphitic (sp2) carbon nature of carbon dots [43]. In Fig. 1e, the FTIR spectrum revealed that the characteristic peaks around 3380 cm^–1^, 2927 cm^–1^, 1719 cm^–1^, 1624 cm^–1^, 1407 cm^–1^, 1184 cm^–1^, and 620 cm^–1^ were indicating the presence of –OH, C–H, –CO, C = C, C–N, C–O, and C–OH functional groups, correspondingly. On the other hand, the surface functional groups of PCDs were further validated through XPS analysis. In Additional file 1: Fig. S3, the C1s spectrum of PCDs was reconverted into four peaks at 284.9 eV, 285.4 eV, and 286.4 eV, corresponding to C–C/C = C, C–O/–N, and C = O bonds [62]. The N1s spectrum revealed two peaks at 400.1 eV and 400.8 eV (Additional file 1: Fig. S4), corresponding to C–N and C–NH bonds [63]. Further, the O1s spectrum showed two peaks at 531.2 eV and 532.6 eV (Additional file 1: Fig. S5), attributed to C = O and C-O bonds [43].

### Optical properties of PCD

**Fig. 2:**
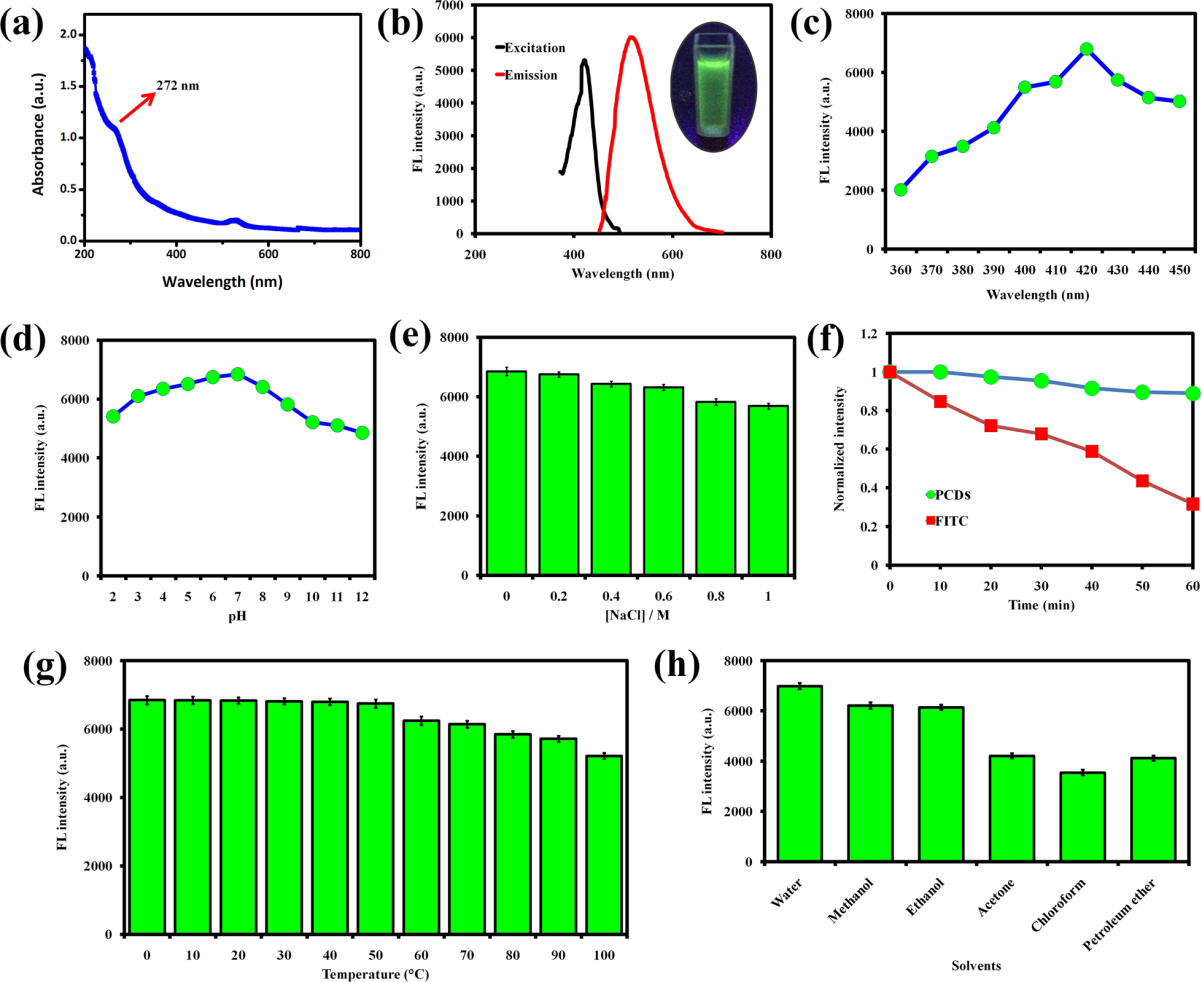
Graph showing the (**a**) UV-Vis absorbance and (**b**) photoluminescence spectrum of the prepared PCDs. The graphs represent the photoluminescence intensity of PCDs under (**c**) different excitation wavelengths, (**d**) pH conditions, and (**e**) salt concentrations. (**f**) Photoluminescence stability of PCDs in photoleaching under UV-irradiation. The graphs represent the photoluminescence intensity of PCDs under (**g**) different temperatures and (**h**) solvents

In Fig. 2a, The UV–Vis absorption spectra revealed a peak at ~ 272 nm, that was attributed to the π–π* transition state of graphitic (sp^2^) carbon [64]. Notably, the photoluminescence property of PCDs was examined at different wavelength. The maximum emission (Em_520nm_) of green photoluminescence was observed at Ex_420 nm_ (Fig. 2b) and it showing an excitation wavelength-dependent emission (Fig. 2c). The excitation-dependent photoluminescence emission was related to the nanoscale diameter of the particles and polydispersity in PCDs with a band gap distribution [65, 66]. It also related with the surface adsorbed functional groups of the PCDs [67]. Quantum yield of the sour apple-derived PCDs were measured to be 26.8%. The stability of the PCDs was further examined at various pH condition, different ionic concentrations, temperatures, and diverse solvents. In Fig. 2d, the results have proved that the FL intensity was unwavering throughout a pH range of 2 to 12 as well as increasing the ionic strength (Fig. 2e). PCDs also has invariable photoluminescence up to 1 h of UV irradiation, which is better than the standard dye, fluorescein isothiocyanate (FITC), proving the photo-stability of PCDs (Fig. 2f). As the temperature rises, the PCD's photoluminescence intensity decreases, which indicates that the green emission of PCDs is temperature sensitive (Fig. 2g). Moreover, PCDs has better photoluminescence in water, methanol, and ethanol, compared to other solvents (Fig. 2h). Hence, we suggest the PCDs for bioimaging application with water or methanol or ethanol solvent for better imaging.

### Non-toxic and biocompatible nature of PCDs

**Fig. 3:**
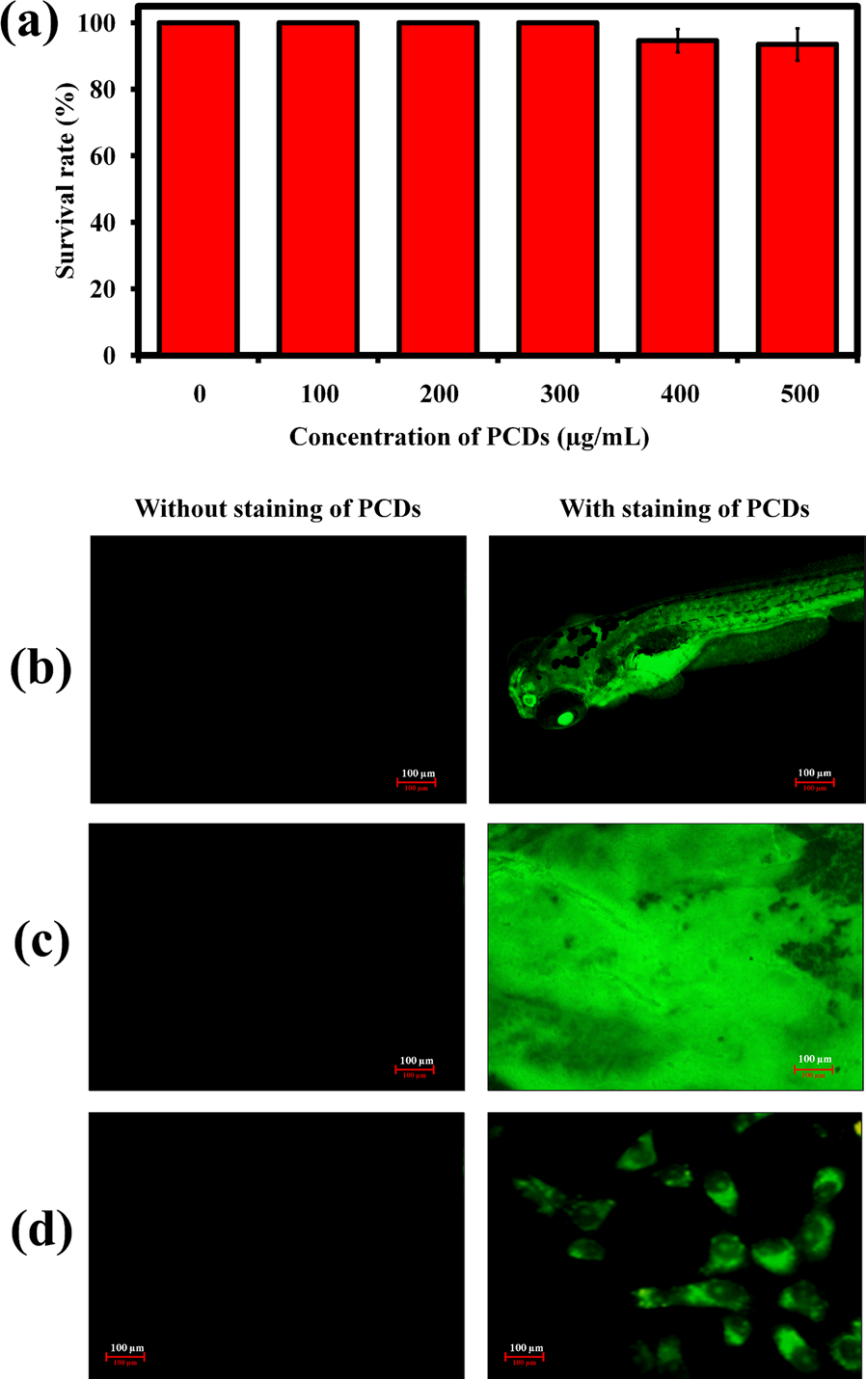
**a** Graph showing the survival percentage of zebrafish larvae in the presence of different concentration of PCDs after toxicity assessment after 96 h. Data is represented as mean ± SD. The in vivo fluorescence ability of synthesized PCDs for bioimaging application in (**b**) zebrafish larvae, (**c**) bacterial biofilms, and (**d**) Vera cells

Before the PCDs employed for bio-imaging application, toxicity level was evaluated using toxicity study with zebrafish model. Due to their similarities to mammalian developmental toxicity pathways, zebrafish have the potential to disclose them as a toxicology model [68]. As a result, zebrafish provides a solid foundation for assessing the toxicity of tested compounds/materials [69]; [70]. The purpose of the toxicity test is to determine whether the synthesized PCDs are suitable for bioimaging applications and to see if utilising them as a bioimaging probe has any potentially harmful side effects [71]. Interestingly, the prepared PCDs did not show any significant mortality to zebrafish larvae up to 500 µg/mL concentration (Fig. 3a), demonstrating the biocompatible nature of PCDs.

### Bioimaging application of PCDs

Bioimaging is a fast growing research field, because of its usefulness in early diagnosis [72], monitoring [73], and imaging-related interventions for existence disorders [74], and also it offers a useful tool for probing cellular elements [75]. Hence, the prepared PCDs were exploited as a probe for fluorescence microscopic imaging with zebrafish larvae as a model to establish the in vivo bio-imaging applicability. After incubation, both clear and strong fluorescence imaging for zebrafish larvae stained with PCDs were observed in the present study (Fig. 3b). It is apparent that the cellular elements in zebrafish larvae had a consistent staining with brilliant luminescence and clear visualization under the excitation of 420 nm, without causing any deleterious effects. Similarly, the PCDs-stained bacterial biofilms of *Staphylococcus aureus* (Fig. 3c) as well as Vero cells (Fig. 3d) showing admirable fluorescence imaging of its structures under a fluorescence microscope. As a conclusion, the result recommends that the PCDs could be executed securely in bioimaging applications.

### PCDs hinders UHMWPE-induced osteoclastogenesis in vitro

**Fig. 4:**
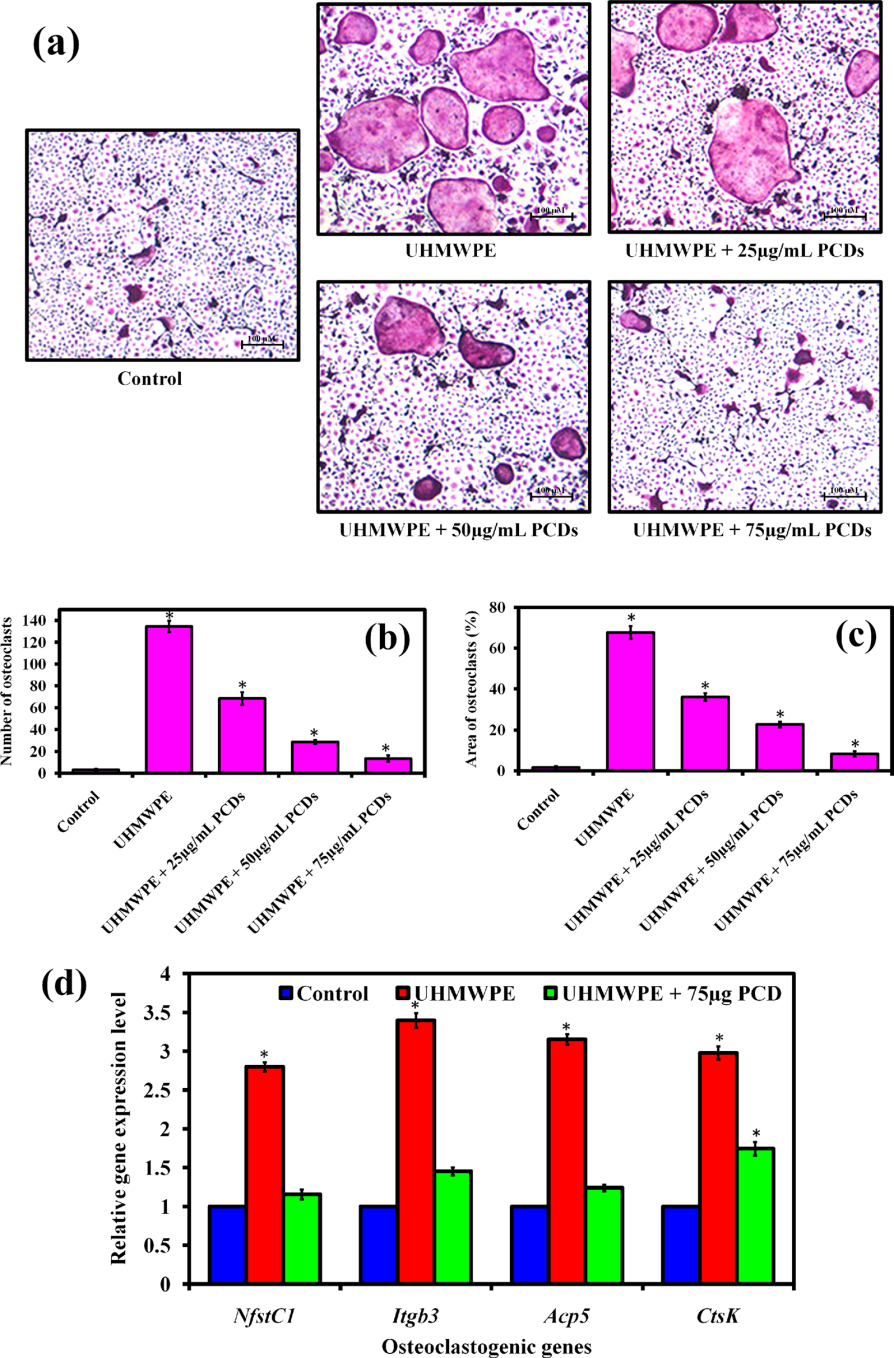
**a** Tartrate-resistant acid phosphatase (TRAP) staining images reveal the effect of PCDs treatment on UHMWPE-induced osteoclast differentiation at different concentrations. Herein, the TRAP-positive cells with more than three nuclei, which were counted to be usual osteoclasts. The graphs representing the (**b**) number of osteoclasts, (**c**) their area of percentage, and **(d)** relative osteoclastogenesis genes in the experimental groups. The number of osteoclasts was counted using ImageJ software. Data are represented as mean ± SD; * indicates P < 0.05 compared with normal control and # indicates P < 0.05 compared with the UHMWPE group to PCD-treated groups

PCDs are innovative new nanomaterials that have recently been applied in medical and therapeutic purposes [76]. In this study, the synthesized PCDs were employed for the first time to treat UHMWPE-induced periprosthetic osteolysis for septic prosthetic loosening in mouse calvarial model. Before the in vivo study, the action of PCDs on osteoclastogenesis was studied in vitro to elucidate the mechanisms by which PCDs inhibited wear particle-induced osteolysis. It is well understood that osteoclasts play an important role in wear particle-induced osteolysis [26]. BMMs are important activator immune cells in inflammatory osteolysis [77]. Several reports revealed that UHMWPE-particles promote the development and functioning of BMMs in the periprosthetic tissues surrounding joint replacements [78]. Therefore, the inhibitory potential of synthesized PCDs on osteoclasts formation from BMMs was examined using TRAP staining method. Hence, BMMs were grown with M-CSF and UHMWPE particle in the absence or presence of increasing concentrations of PCDs for 7 days. In the control group, many TRAP-positive multinucleated osteoclasts developed (Fig. 4a). PCDs treatment, on the other hand, suppressed osteoclast development in a dose-dependent manner. The result revealed that the number of osteoclasts was reduced to the level of 81.2% in the PCDs treated group (75 µg/mL) compared to UHMWPE-induced group (Fig. 4b, c) as well as the relative osteoclastogenesis genes were also decreased (Fig. 4d). Simultaneously, PCDs did not cause toxicity in BMMs at concentrations up to 200 µg/mL (Additional file 1: Fig. S6), as validated by the cell viability experiment using the CCK-8 method. Overall, these results conclude that the PCDs effectively hinder the UHMWPE-induced osteoclastogenesis without having any cytotoxic effect on BMMs.

### PCDs inhibits F-actin ring formation and bone resorption

**Fig. 5:**
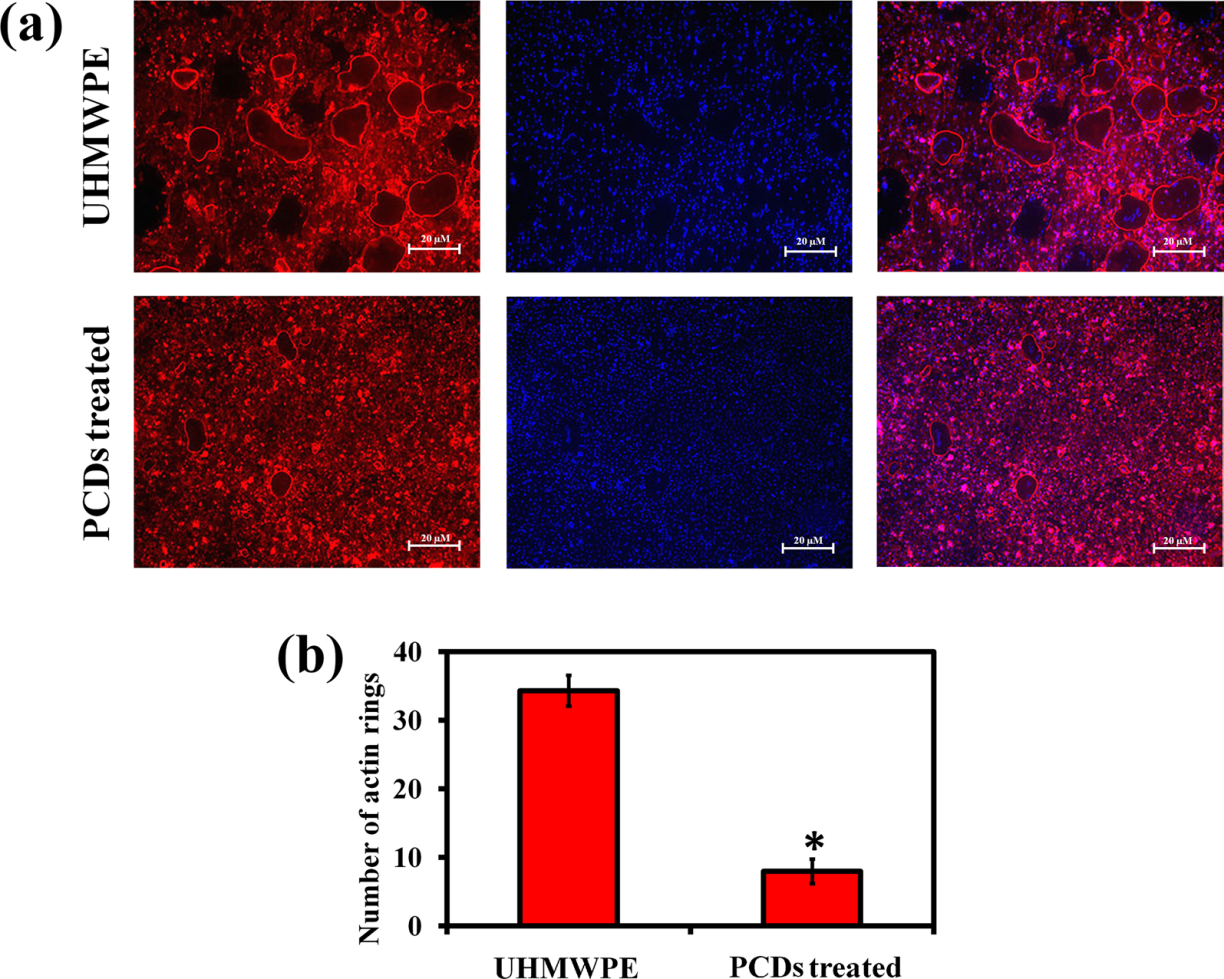
**a** Representative fluorescence microscope of phalloidin and DAPI-stained osteoclast cells for assessing the effect of PCDs on UHMWPE-induced F-actin ring formation in vitro. **b** Graph representing the relative number of F-actin positive cells measured by Image-J software

Since the development of mature F-actin rings is required for osteoclast bone resorption [79], F-actin ring staining was performed to investigate the inhibitory action of PCDs on F-actin ring development. In Fig. 5a, the staining image showed the F-actin ring development with the typical podosomal closure of aster-like ring structure of osteoclasts in UHMWPE group. Conversely, the presence and abundance of F-actin rings dropped dramatically in the cells treated with PCDs at 75 µg/mL concentration (Fig. 5b), indicating that PCDs inhibited F-actin ring development.

**Fig. 6:**
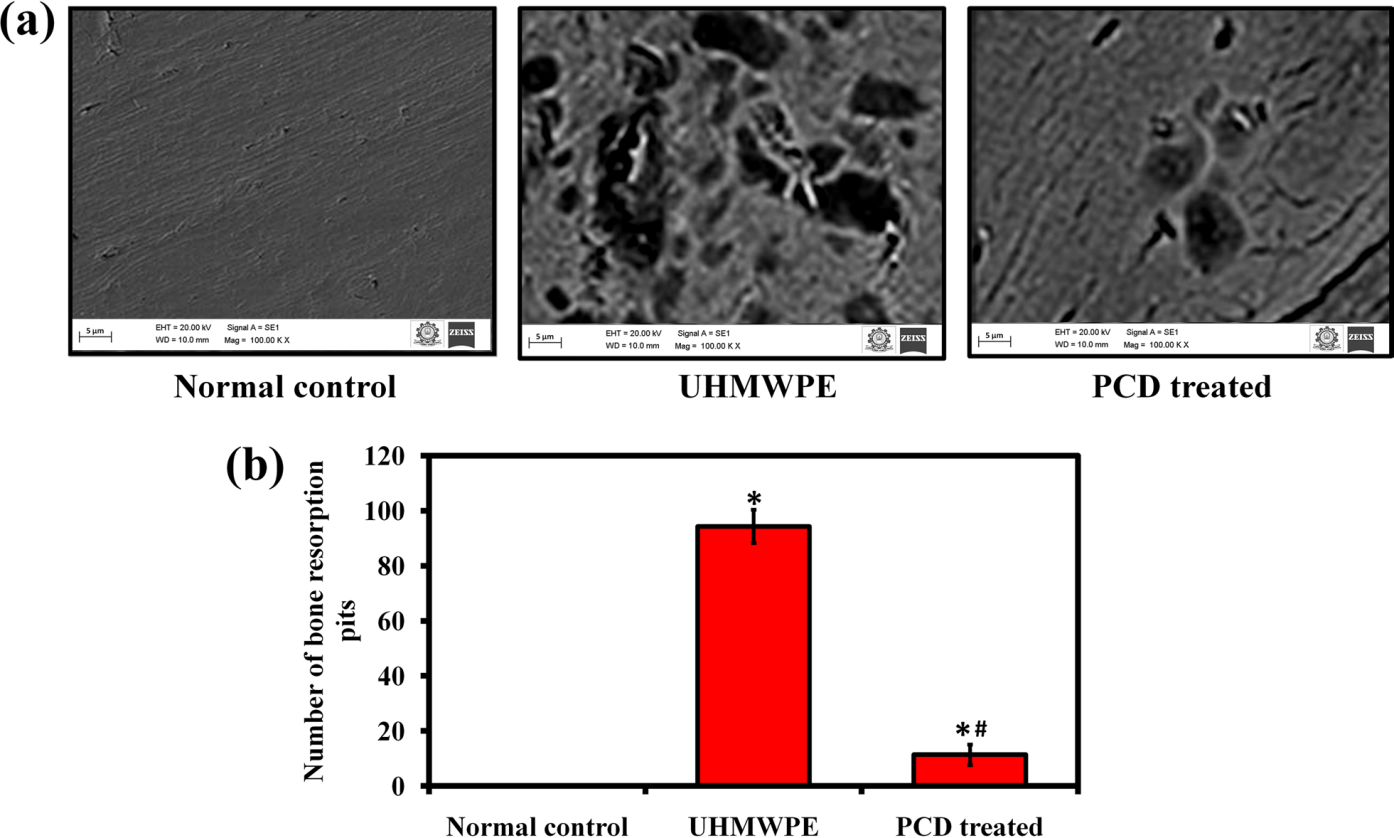
**a** Representative images of UHMWPE-induced bone resorption and the rescue action of PCDs in the treated group observed via SEM analysis. **b** The graph illustrating the number of bone resorption pits in the PCDs group compared to UHMWPE group. The number of resorption pits was analyzed using Image-J software. Data are represented as mean ± SD; * indicates P < 0.05 compared with normal control and # indicates P < 0.05 compared with the UHMWPE group to PCDs-treated group

Further, the preventive potential of PCDs on UHMWPE-induced osteoclastic bone resorption was assessed using SEM analysis. The synthesized PCDs can prevent osteoclast development; we found that it will also diminish osteoclastic bone resorption (Fig. 6a). The UHMWPE group had irregular erosion pits that were widely scattered on the osteo assay plate. In the PCDs treated group, the number of bone resorption pits lowered to the level of 87.98% compared to UHMWPE group (Fig. 6b); even very few resorption pits identified in the PCDs treated group. These results showed that PCDs inhibited osteoclast F-actin ring growth and bone resorption in vitro, motivating us to do animal studies for osteolysis.

### PCDs inhibits UHMWPE-induced ROS-mediated osteoclastogenesis

**Fig. 7:**
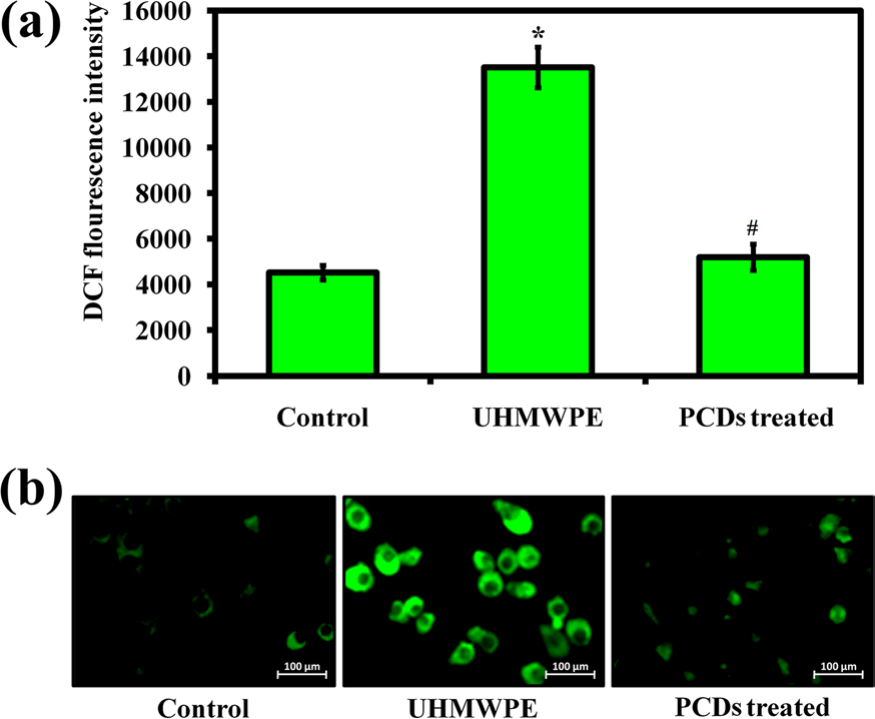
**a** Graph indicates the DCF fluorescence intensity in the experimental groups, symbolizing the UHMWPE-induced ROS intensity in the experimental groups during osteoclastogenesis. Data are represented as mean ± SD; Error bars stand for how much errors are happened during the experiments. * indicates P < 0.05 compared with normal control and # indicates P < 0.05 compared with the UHMWPE group to PCD-treated group. **b** Representative fluorescence microscope images of UHMWPE-induced ROS intensity in osteoclasts

Implant-based particle debris, including UHMWPE, elicits a systemic immunological response and attracts monocytic cells to phagocytoze granules, resulting in the induction of ROS stress and augmented the secretion of pro-inflammatory cytokines, including TNF-α, IL-1, IL-6, and IL-8, all of which make a significant participation to osteolysis [80]. Oxidative stress caused by ROS is one of the factors that cause osteoclasts to accelerate bone resorption [20]. Therefore, the inhibitory potential of PCDs against UHMWPE-induced ROS-stress in BMMs was analyzed using DCFH-DA staining. Before the experiment, the antioxidant nature of PCDs was primarily assessed using DPPH assay. In Additional file 1: Fig. S7, the DPPH assay result revealed that the PCDs scavenged the DPPH free radicals to the level of 96.55% at 75 µg/mL concentration, illustrating the antioxidant nature of PCDs. Similarly, the PCDs effectively scavenged other biological ROS radicals such as H_2_O_2_, •OH, and O2• − radicals to the level of 84.33%, 87.82%, and 82.1%, respectively (Additional file 1: Fig. S8). As expected, the PCDs efficiently scavenged the UHMWPE-induced ROS generation at 75 µg/mL concentration (Fig. 7a). The fluorescence microscopic images showed that the reduced intensity of ROS in the treatment group indicating the inhibitory potential of PCDs on UHMWPE-induced ROS generation (Fig. 7b). The result revealed that the prepared PCDs have the ability to avoid ROS-mediated osteoclastogenesis.

### PCDs suppressed the Chemerin/ChemR23 signaling and NFATc1 pathway

**Fig. 8:**
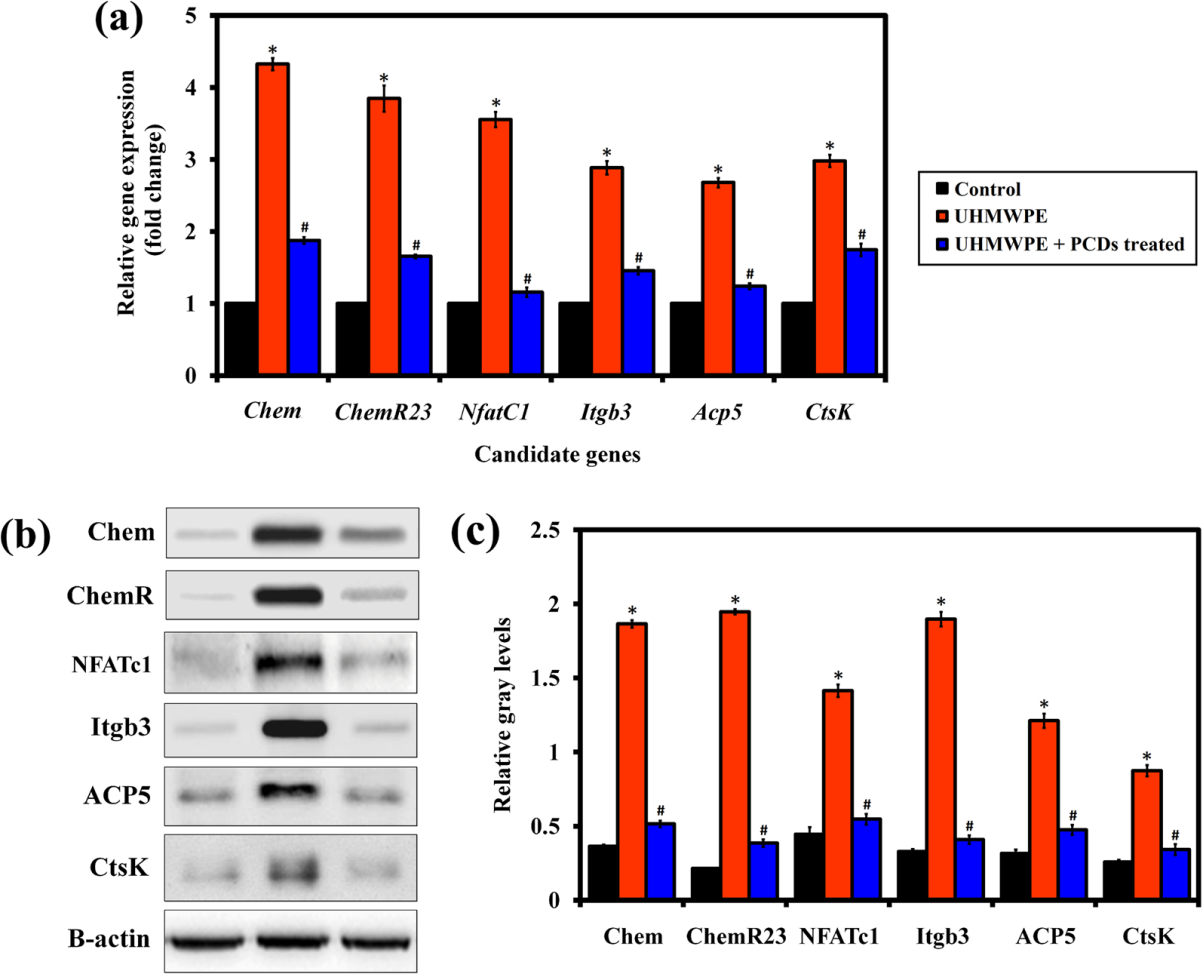
PCDs suppressed the UHMWPE-induced osteoclastogenesis through Chemerin/ChemR23 signaling and NFATc1 pathway. **a** Graph representing the relative expression level of osteoclastogenesis-related genes in the experimental groups, which were analyzed using qPCR analysis. **b** Representative western blot images of proteins and (**c**) their relative gray levels corresponding to Chem, ChemR23, NFATc1, Itgb3, ACP5, CtsK, and β-actin. Data are represented as mean ± SD; Error bars stand for how much errors are happened during the experiments. * indicates P < 0.05 compared with normal control and # indicates P < 0.05 compared with the UHMWPE group to PCD-treated group

Chemerin/ChemR23 signaling is important in the actions of UHMWPE-particles on the equilibrium of osteogenic and osteoclastogenic development, which alters the pattern of osteogenesis and leads to bone resorption [17]. Furthermore, Chemerin/ChemR23 signalling increases joint inflammation and also involved in the molar teeth development [81]. Subsequently, nuclear factor of activated T-cells cytoplasmic-1 (NFATc1) is a nuclear transcriptional factor synchronized by NF-κB, which is necessary for initiating osteoclastogenesis [82, 83]. Also, NFATc1 is necessary for Chemerin/ChemR23-mediated stimulation of osteoclastogenesis through UHMWPE-particles [16]. Altogether, the effect of PCDs on expression level of these abovementioned genes was examined using qPCR analysis and western blot analysis. As expected, the results revealed that the UHMWPE-particles increased the gene expression level of *Chem* (*Rarres2*), *Chem23*, and the osteoclastogenic transcription factor NFATc1with improved expression of the osteoclast specific markers such as *Acp5, Ctsk*, and *Itgb3* (Fig. 8a). This result was highly similar with previous studies [17, 84]. On the other hand, these osteoclastogenesis-related genes were significantly downregulated in the PCDs treated group, when compared to UHMWPE group. In Fig. 8b, c, western blot results revealed that the phosphorylation of Chem, Chem23, NFATc1, ACP5, Ctsk, and Itgb3 was reduced in PCDs treated group, which was consistent with the qPCR results (Fig. 8a). Overall, these findings concluded that PCDs could reduce UHMWPE-induced Chemerin/ChemR23 signaling and NFATc1 pathway during osteoclastogenesis.

### PCDs also improving the SIRT1 expression

**Fig. 9:**
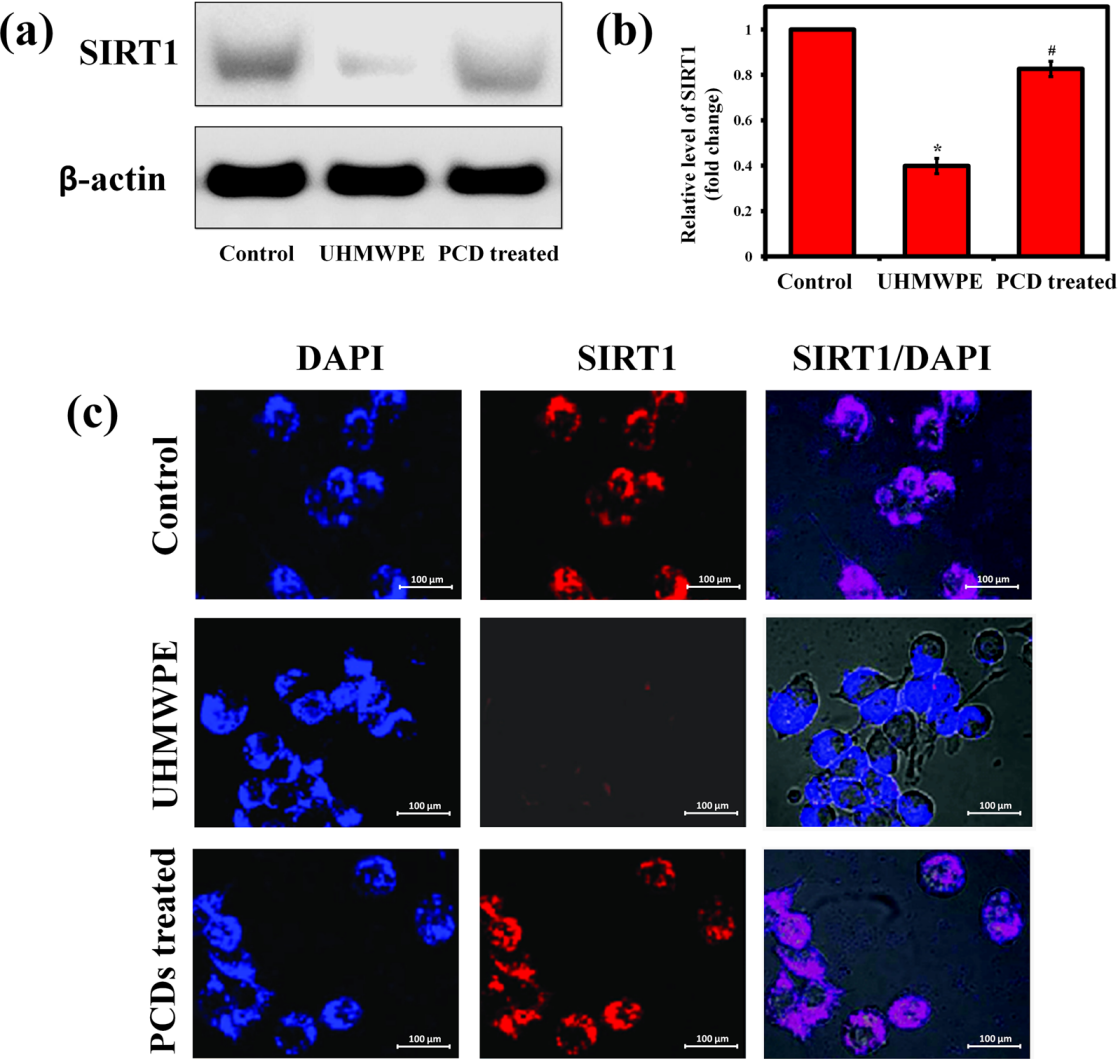
PCDs also improve the expression of SIRT1 for reducing the UHMWPE-induced osteoclastogenesis. **a** Representative western blot images of SIRT1 expression and (**b**) their relative gray level in the experimental groups. **c** Representative fluorescence microscope images of SIRT1/DAPI stained osteoclasts, which showing the expression level of SIRT1 in the form of red flourescence. Data are represented as mean ± SD; * indicates P < 0.05 compared with normal control and # indicates P < 0.05 compared with the UHMWPE group to PCD-treated group

Previous study has reported that the upregulation of SIRT1 decreases murine osteoclast progenitor proliferation, which lowers osteoclastogenesis [85]. SIRT1 activation modulates the expression of p53 and PPAR, causing a reduction in osteoclastogenesis with increased osteoblast differentiation [86]. Hence, the expression level of SIRT1 was measured through western blot to witness the rescue effect PCDs on UHMWPE-induced pro-inflammatory response associated with SIRT1 pathway. Initially, we noticed that when macrophages were treated with UHMWPE, the protein level of SIRT1 was decreased than in untreated control (Fig. 9a). Conversely, the PCDs treatment greatly increased SIRT1 expression that had been reduced by UHMWPE particle (Fig. 9b). Since the BMMs are interacting with UHMWPE-wear debris and secrete different inflammatory cytokines, namely TNF-α, IL-1β, and IL-6 [87], the effect of PCDs treatment against UHMWPE-induced inflammatory cytokines secretion was analyzed. As expected, the increase in TNF-α, IL-1β, and IL-6 caused by UHMWPE was reduced by PCDs, confirming these findings (Additional file 1: Fig. S9). Altogether, our findings imply that PCDs increases SIRT1 expression while decreasing UHMWPE-induced inflammatory cytokine release, resulting in anti-osteolysis activity.

### Inhibitory effect of PCDs in UHMWPE-induced osteolysis in mouse calvarial model

**Fig. 10:**
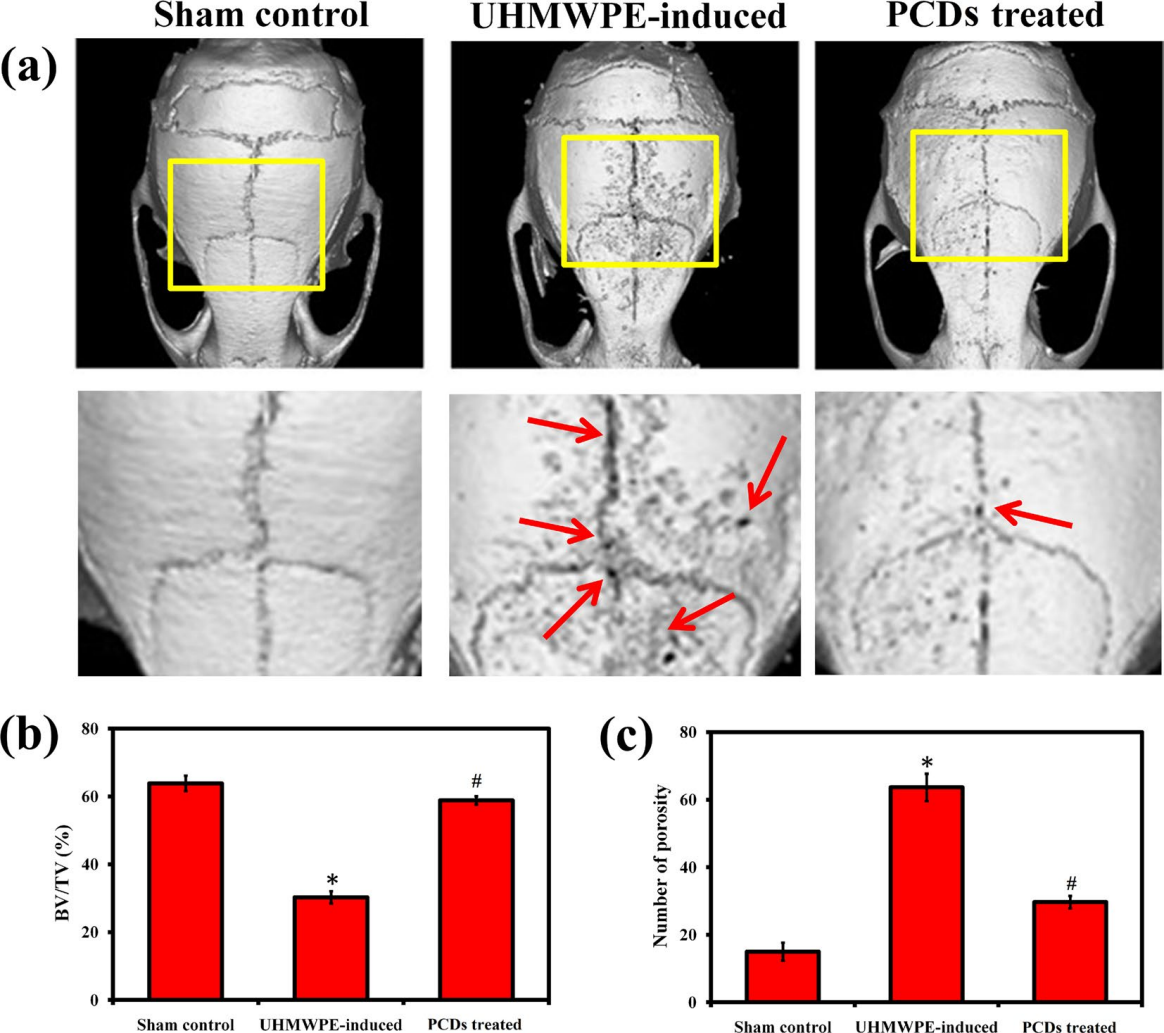
Inhibitory potential of PCDs in UHMWPE-induced osteolysis in mouse calvarial model. **a** Showing the representative micro-CT 3D image of PCD-treated calvaria. The red colour arrow indicates the bone loss in mouse calvaria. Graphs illustrating the (**b**) bone volume/total volume (BV/TV) and (**c**) total porosity of experimental group. Data are represented as mean ± SD; *P < 0.05 compared with sham control and #P < 0.05 compared with the UHMWPE group

To evaluate the inhibitory effect of PCDs on periprosthetic osteolysis, we utilized a mouse calvaria model with UHMWPE-particles to cause symptomatic osteolysis. Therefore, we have administered PCDs (7.5 mg/kg) into the bone resorption sites after the surgical incision had healed. After 14 days, the bone resorption and its treatment with PCDs were assessed using micro-CT image reconstruction. As seen in Fig. 10a, the UHMWPE group had a lot more bone resorption when compared to the sham group; whereas, the PCDs group exhibited a considerable reduction in bone resorption. In Fig. 10b, bone parameter analysis revealed that calvaria destruction was much higher in the UHMWPE group compared to the sham group, whereas PCDs treatment greatly reduced the BS/BV and total porosity. Overall, this part concludes that PCDs possesses inhibitory action on UHMWPE-induced osteolysis in vivo, according to our findings. The suppression of chemerin/ChemR23 signaling and SIRT1 pathway may be responsible for this inhibitory action of PCDs on UHMWPE-induced osteolysis.

## Conclusion

In this study, we present a biocompatible and green photoluminescent carbon dots for novel functionality of lessening UHMWPE particle-induced osteolysis, as well as bioimaging application. Due to the commendable fluorescence emission inside the hosts, the synthesized PCDs were recommended for bioimaging usage. The PCDs effectively inhibited UHMWPE wear particle-induced osteoclastogenesis, F-actin ring pattern, and bone resorption in vitro. Furthermore, the PCDs decreased the ROS stress caused by UHMWPE as well as the release of pro-inflammatory cytokines such as TNF-α, IL-1β, IL-6, and IL-8. The qPCR and western blot results found that PCDs reduced the UHMWPE particle-induced osteolysis in mouse calvaria via decreasing chemerin/ChemR23 signaling and NFATc1 pathway, as well as upregulating SIRT1 expression. These findings showed that PCDs could be used to treat symptomatic osteolysis and osteoclast-mediated illnesses. The present study suggests that the synthesized PCDs could be the promising agent to control osteoclastogenesis. These findings might pave the way for a novel approach to reducing UHMWPE wear particle-induced aseptic prosthesis loosening in future.

## Supplementary Information

**Additional file 1: Fig. S1.** Phytochemical analysis of sour apple peel extract using GC-MS analysis. **Fig. S2** XRD spectrum of sour apple peel extract. **Fig. S3.** C1s XPS spectrum of as-prepared PCDs. **Fig. S4** N1s XPS spectrum of as-prepared PCDs. **Fig. S5** O1s XPS spectrum of as-prepared PCDs. **Fig. S6** Graph representing the cell viability of BMMs in the presence of different concentrations of PCDs. The cell viability was measured by CCK-8 assay. Values are presented as means ± SD from three independent experiments. * = P < 0.05 compared to control. **Fig. S7** Graph representing the DPPH scavenging activity, which reveals the antioxidant property of synthesized PCDs. Values are presented as means ± SD from three independent experiments. * = P < 0.05 compared to control. **Fig. S8** Graph representing the scavenging activity of PCDs on other biological ROS radicals such as H2O2, •OH, and O2•− radicals. Values are presented as means ± SD from three independent experiments. * = P < 0.05 compared to control. **Fig. S9** The expression of pro-inflammatory cytokines (TNF-α, IL-1β, and IL-6) in BMMs that treated with PCDs before being stimulated with UHMWPE for 3 days. Values are presented as means ± SD from three independent experiments. * = P < 0.05 compared to control; # = P < 0.05 compared to UHMWPE-treated group. **Table S1** Sequences of primers used in the present study.

## Data Availability

The authors confirm that the data supporting of the present study are available within the article and its supplementary material.
