# Supplementary material for: Photoluminescent carbon dots (PCDs) from sour apple: a biocompatible nanomaterial for preventing UHMWPE wear-particle induced osteolysis via modulating Chemerin/ChemR23 and SIRT1 signaling pathway and its bioimaging application
Source: J Nanobiotechnology. 2022 Jun 27;20:301. doi: 10.1186/s12951-022-01498-3 (PMC9235131; doi:10.1186/s12951-022-01498-3)
Supplement: Supplementary file 1 — Additional file 1: Fig. S1. Phytochemical analysis of sour apple peel extract using GC-MS analysis. Fig. S2 XRD spectrum of sour apple peel extract. Fig. S3. C1s XPS spectrum of as-prepared PCDs. Fig. S4 N1s XPS spectrum of as-prepared PCDs. Fig. S5 O1s XPS spectrum of as-prepared PCDs. Fig. S6 Graph representing the cell viability of BMMs in the presence of different concentrations of PCDs. The cell viability was measured by CCK-8 assay. Values are presented as means ± SD from three independent experiments. * = P < 0.05 compared to control. Fig. S7 Graph representing the DPPH scavenging activity, which reveals the antioxidant property of synthesized PCDs. Values are presented as means ± SD from three independent experiments. * = P < 0.05 compared to control. Fig. S8 Graph representing the scavenging activity of PCDs on other biological ROS radicals such as H2O2, •OH, and O2•− radicals. Values are presented as means ± SD from three independent experiments. * = P < 0.05 compared to control. Fig. S9 The expression of pro-inflammatory cytokines (TNF-α, IL-1β, and IL-6) in BMMs that treated with PCDs before being stimulated with UHMWPE for 3 days. Values are presented as means ± SD from three independent experiments. * = P < 0.05 compared to control; # = P < 0.05 compared to UHMWPE-treated group. Table S1 Sequences of primers used in the present study. [file 12951_2022_1498_MOESM1_ESM.pdf]

Supplementary Material

Supplementary Figures

Fig. S1 Phytochemical analysis of sour apple peel extract using GC-MS analysis.

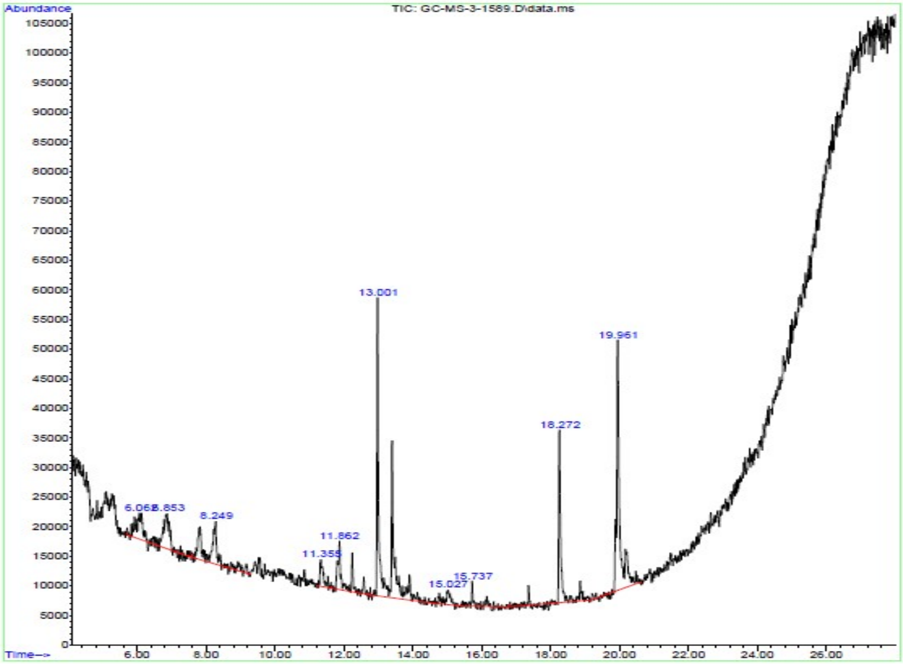

Peak No.	RT (in min)	Compound Name	Area (%)
1	6.064	Cyclohexa-2,5-diene-1,4-dione, 2-methyl-5-(4-morpholinyl)-	5.91
2	6.853	Cyclopropane, 1-methyl-2-(3-methyl pentyl)-	5.83
3	8.249	Malic acid	18.74
4	11.355	3-Methylmannoside	2.88
5	11.862	Propenone, 3-(2-benzoxazolylthio)-1-phenyl-	10.97
6	13.001	Citric acid	20.34
7	15.027	1,2,4-Triazol-3-amine, 5-(1,3,5-tr imethyl-4-pyrazolyl) amino-	2.60
8	15.737	3-Hexyn-2-ol	1.86
9	18.272	Hexadecanoic acid, methyl ester	11.21
10	19.961	9-Octadecenoic acid	19.66

Fig. S2 XRD spectrum of sour apple peel extract.

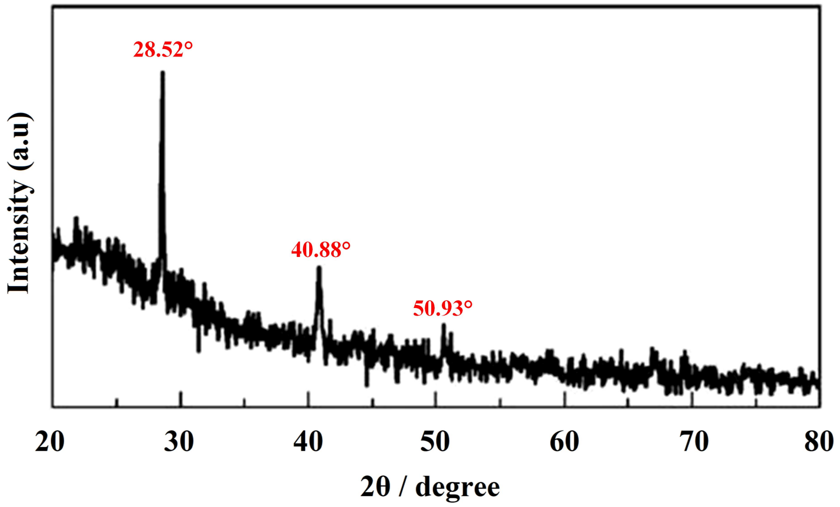

Fig. S3 C1s XPS spectrum of as-prepared PCDs.

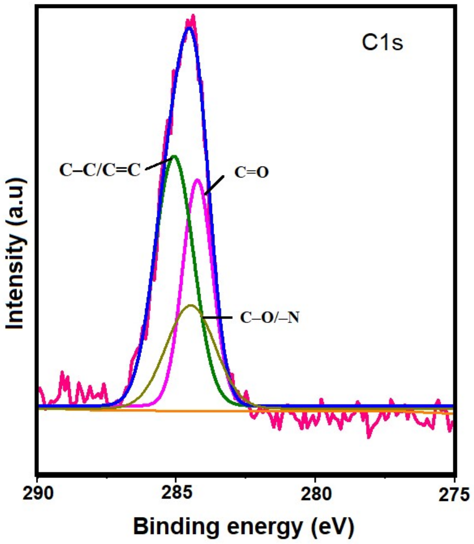

Fig. S4 N1s XPS spectrum of as-prepared PCDs.

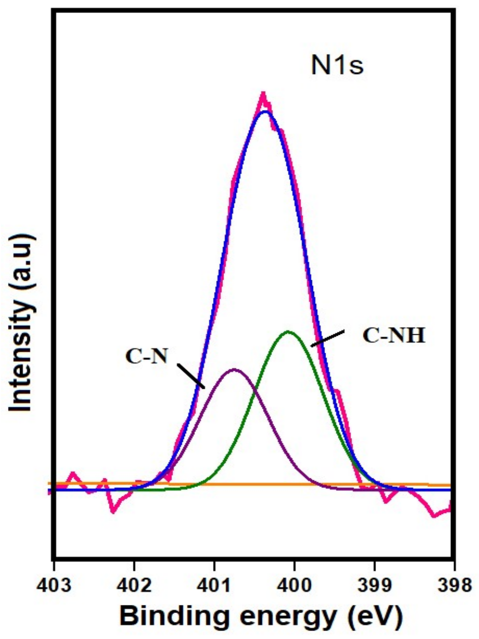

Fig. S5 O1s XPS spectrum of as-prepared PCDs.

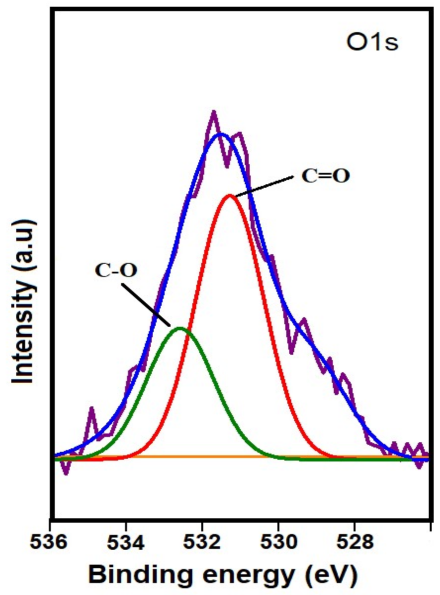

Fig. S6 Graph representing the cell viability of BMMs in the presence of different concentrations of PCDs. The cell viability was measured by CCK-8 assay. Values are presented as means ± SD from three independent experiments. * = $P < 0.05$ compared to control.

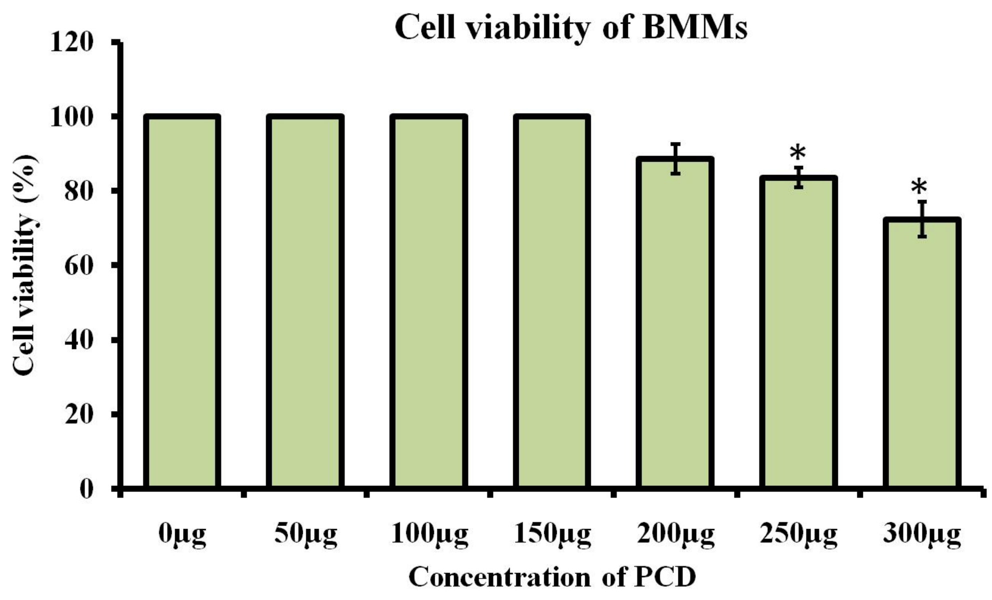

Fig. S7 Graph representing the DPPH scavenging activity, which reveals the antioxidant property of synthesized PCDs. Values are presented as means ± SD from three independent experiments. * = $P < 0.05$ compared to control.

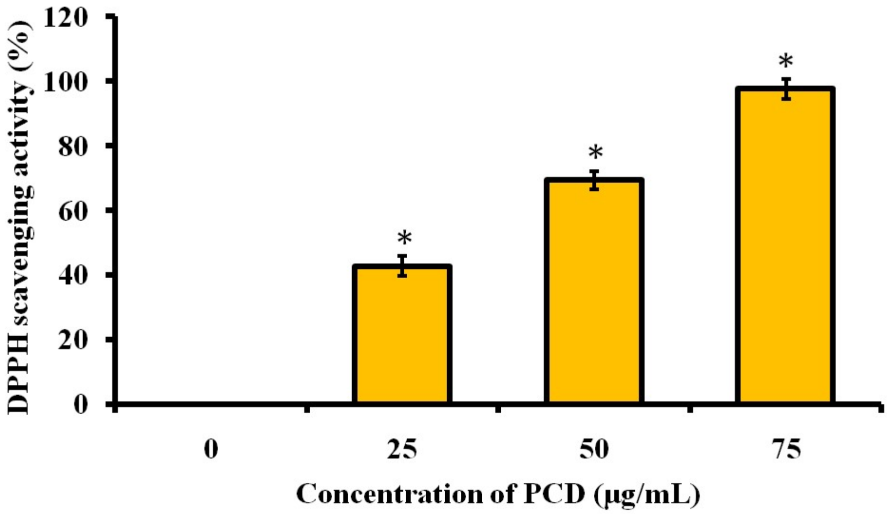

Fig. S8 Graph representing the scavenging activity of PCDs on other biological ROS radicals such as H_2O_2, •OH, and O2•− radicals. Values are presented as means ± SD from three independent experiments. * = P < 0.05 compared to control.

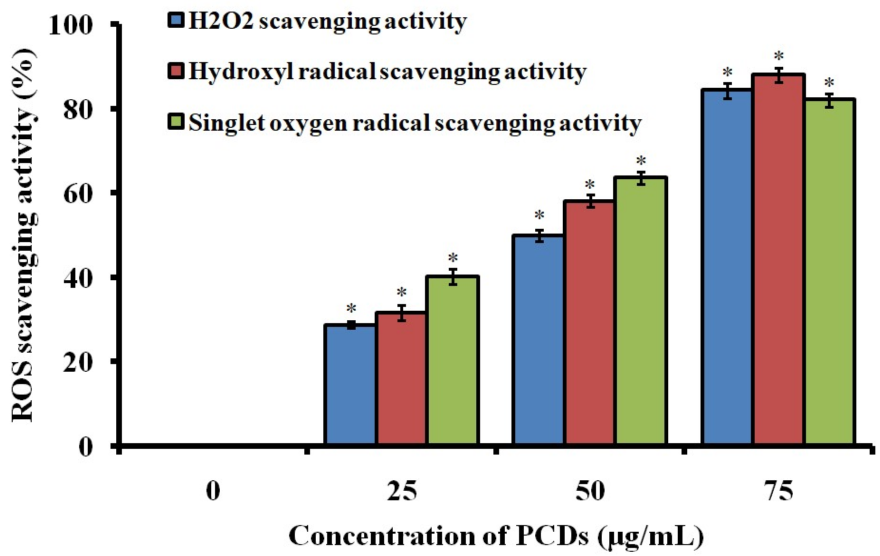

Fig. S9 The expression of pro-inflammatory cytokines (TNF-α, IL-1β, and IL-6) in BMMs that treated with PCDs before being stimulated with UHMWPE for 3 days. Values are presented as means ± SD from three independent experiments. * = P < 0.05 compared to control; # = P < 0.05 compared to UHMWPE-treated group.

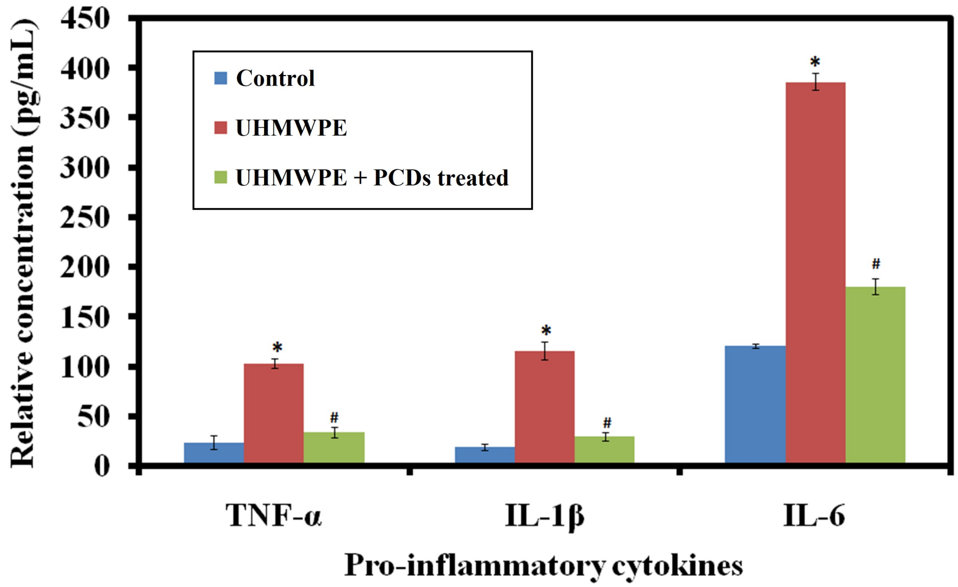

Supplementary Tables

Table S1 Sequences of primers used in the present study

Gene	Primer Sequence	
	Forward primer	Reverse primer
ChemR23	ACCACTGGGTGTTCGGGAAGG	ACGGAGATGCAGCGGTCAAA
Rarres2	CCCTGAGAACCAAATAAGCCCT	AGCCTGGAGTTGAAAGTCTCTG
NfatC1	TGGTTGAGATACCACCTTTCCG	AGAGTTACCATTGGCAGGAAGG
Acp5	GAAAGTCAAGGGAGTGGCAGGG	ACCTCAGGGCCTTTGTCCTCA
Ctsk	GTTGTATGTATAACGCCACGGC	CTCTCTTCAGGGCTTTCTCGTT
Itgb3	ACATCACACATGCTAGGCAAGA	GGTCAACATTCCAGCCTCCTTA
Runx2	ACCCATTGGTATCTGCCATTGT	AGGAAGCCACACTTAGGGATTG
Alp	ATATCGACGTGATCATGGGTGG	GATGAGATCCAGGCCATCTAGC
Osx	TCCCCTAGGTTATCTCCTTGCA	TAGGAACTAGGCACTGGCAAAG
**β-actin**	GGTGAAGGTCGGTGTGAACG	CTCGCTCCTGGAAGATGGTG
